# Enteral Nutrition Support in Burn Care: A Review of Current Recommendations as Instituted in the Ross Tilley Burn Centre

**DOI:** 10.3390/nu4111554

**Published:** 2012-10-29

**Authors:** Kathryn L. Hall, Shahriar Shahrokhi, Marc G. Jeschke

**Affiliations:** Ross Tilley Burn Centre, Sunnybrook Health Sciences Centre, 2075 Bayview Avenue, Toronto, Ontario, M4N 3M5, Canada; Email: Kathryn.Hall@sunnybrook.ca (K.L.H.); shar.shahrokhi@sunnybrook.ca (S.S.)

**Keywords:** burns, enteral nutrition, antioxidants

## Abstract

Failure to adequately address the increased levels of inflammatory mediators, catecholamines and corticosteroids central to the hypermetabolic response post burn injury can lead to catastrophic results. One of the most important perturbations is provision of adequate and early nutrition. The provision of the right balance of macro and micronutrients, along with additional antioxidants is essential to mitigating the hypermetabolic and hypercatabolic state that results following a burn injury. As it is now widely accepted that enteral feeding is best practice for the burn population research has been more closely examining the individual components of enteral nutrition support. Recently fat to carbohydrate ratios, glutamine and antioxidants have made up the balance of this focus. This paper provides a review of the most recent literature examining each of these components and discusses the practices adopted in the Ross Tilley Burn Centre at Sunnybrook Health Sciences Centre.

## 1. Introduction

As is widely reported in the literature, the early initiation of enteral nutrition support in the burn population is of utmost importance for survival [1,2]. The composition of this enteral nutrition support is equally important. The provision of the right balance of macro and micronutrients, antioxidants and energy is essential for mitigating the hypermetabolic and hypercatabolic state that results following a burn injury [1,2]. 

The Ross Tilley Burn Centre at Sunnybrook Health Sciences Centre is an American Burn Association verified burn centre with 250 annual admissions and 700–900 patients followed in its out-patient clinics. The Ross Tilley Burn Centre only admits patients over >16 years of age, 70% of whom have burns with greater than 20% total body surface area involvement. 

This paper provides a review of the most recent literature examining each of the above-mentioned nutritional components and discusses the practices adopted in the Ross Tilley Burn Centre. The adopted practices discussed within this paper have contributed to decreased infection rates, better graft take and a mortality rate of less than 2% within the centre.

## 2. Review of Metabolic Response to Burn

The physiologic response to a burn injury goes well beyond the site of injury and involves changes in the function of the liver, heart, gastrointestinal tract, muscle, bone, kidney and serum levels of catecholamines, corticosteroids and inflammatory cytokines [3,4,5]. These changes can continue to have an effect on metabolic function for upwards of two years post severe burn injury with hypercatabolism persisting for a minimum of 9 months [6] and up to three years post injury [7]. 

The initial hypometabolic “ebb” phase is followed by a hypermetabolic “flow” phase of the stress response which can last from days to weeks. This stress response can be addressed through a number of strategies implemented by the burn team including: surgically through the excision and grafting of the wound; pharmacologically with the provision of catecholamine antagonists, analgesia, and anabolic hormones; and nutritionally with the provision of antioxidants and enteral nutrition support with adequate protein and carbohydrate [1].

The cytokine cascade, catecholamines and corticosteroids are central to the hypermetabolic response post burn injury. The circulating serum levels of catecholamines and corticosteroids elevate by ten to twenty fold post burn and for up to twelve months post burn. This increase in catabolic hormones results in insulin resistance, increased gluconeogenesis, energy consumption, lipolysis and proteolysis. While gluconeogenesis may provide some benefit with the resulting increase in serum glucose levels it is not “protein sparing” and can lead to a multitude of problems including poorer skin graft take, increased incidence of wound infection, and overall poorer rates of morbidity and mortality [1]. 

Failure to address these metabolic changes can have catastrophic results. Prolonged hypercatabolism can lead to fatal cachexia with a weight loss of 40% of admission weight [1,8]. Other events that occur within the context of hypercatabolism include increased rates of bone catabolism, immunosuppression and in children growth retardation that can persist up to one year post injury [1,9]. Lean body mass losses of 10% can lead to immune dysfunction and a loss of 20% can lead to significant impairment with wound healing [1]. 

## 3. Enteral Nutrition and Burns

### 3.1. Importance of Early Post Burn Enteral Feeding

While current evidence remains inconclusive about the benefits of “early” (as defined by feeding in the first 24 h) *vs.* “late” (as defined by more than 24 h post burn) enteral nutrition and as to its impact on length of stay or mortality rates there is reason to believe that early enteral nutrition may positively impact the magnitude of the initial hypermetabolic response [10]. Enteral nutrition is beneficial in maintaining immunity associated with the gut associated lymphoid tissue [11] thus providing even low rates of enteral nutrition early in the admission has a theoretical advantage.

Previous recommendations have included starting enteral nutrition support within six hours at a low rate (approximately ten milliliters per hour) via a post pyloric feeding tube so as to bypass any possible gastric dysmotility and achieving the goal rate for enteral nutrition by day three post injury. This allows for preservation of the gut associated lymphoid tissue, stimulates gut motility and intestinal blood flow in an effort to protect against intestinal hypoperfusion [1]. 

The practice in the Ross Tilley Burn Centre is to place an enteral feeding tube on admission for large burns and initiate enteral nutrition support within 8–12 h of admission with the goal of advancing to and achieving goal infusion rates once the patient is adequately fluid resuscitated. The practice of placing nasojejunal or orojejunal tubes in all patients with greater than 20% total body surface area (TBSA) burns has been adopted in our centre to maximize the delivery of nutrients by decreasing the amount of time during which feeds are held. 

### 3.2. Macronutrient Composition of Post Burn Feeds

#### 3.2.1. Carbohydrate

The provision of adequate amounts of carbohydrates is important for the preservation of lean body mass in the burn population as it spares protein from being used as an energy source [1]. When providing carbohydrate (especially via parenteral route), it is important not to exceed the maximum rate at which glucose can be assimilated in the body (7 g/kg/day) so as not to provide glucose in excess of the rate at which it can be oxidized [12]. When calculating the nutritional requirements of a burn patient it is possible that the amount of carbohydrate theoretically required is higher than the rate at which the body can oxidize it. 

Providing the appropriate amount of carbohydrate is a fine balancing act. The provision of excessive amounts of carbohydrate can have deleterious effects including: hyperglycemia, conversion of glucose to fat, polyuria resulting in poor fluid balance, glucosuria, and excessive carbon dioxide production making ventilation and weaning from ventilator support more difficult [1]. Failure to provide adequate carbohydrate can lead to greater rates of catabolism and loss of lean muscle mass, failure to heal burn wounds, sepsis, infection and death [1,13,14].

Studies have shown that providing severely burned patients with a high carbohydrate, high protein, low fat enteral product can lower the incidence of catabolism and pneumonia when compared to severely burned patients receiving high fat, high protein, low carbohydrate enteral products [15]. These same studies, however, were not able to show conclusive evidence of difference in mortality rates.

#### 3.2.2. Fat

The amount of fat that is provided as part of the enteral diet needs to be carefully determined. There is a vast alteration in the way that fat is metabolized in the body post burn and the provision of excess exogenous sources of fat through propofol and/or enteral or parenteral nutrition can result in significant stress on the liver [1]. The increased breakdown of peripheral fat stores immediately after injury and increased beta oxidation of fat to be used as fuel during the hypermetabolic phase leads to the potential of significant accumulations of fat in the liver [16].

The provision of fat as part of the enteral diet post burn is required to prevent essential fatty acid deficiencies and as such a minimum of 2%–4% of total calories provided needs to be from essential fatty acids [1]. Omega three fatty acids are known to have immune-modulating and anti inflammatory effects on the body that have been associated with improved outcomes after burn injury [1,17]. Conversely, care should be taken to monitor the amount of omega-6 fatty acids provided to the burn patient as these fatty acids are known to metabolize into pro-inflammatory cytokines [1], particularly in patients with underlying chronic diseases such as non-alcoholic fatty liver disease and cardiovascular disease [17]. Providing enteral nutrition that is low in fat is necessary to prevent essential fatty acid deficiencies while decreasing the danger of fat enrichment in the organs, most notably the liver.

The lipid provided in the enteral nutrition formula used in the Ross Tilley Burn Centre is not ideal at 31% of total kilocalories, however, once the additional supplementation of protein and glutamine occurs (as discussed below) this percentage decreases to 27%–28% of kilocalories provided. Lipid and calories provided through propofol is carefully accounted for and the enteral feeding volume is adjusted. While there are enteral formulas with lower lipid contents, they often do not contain adequate amounts of protein and lack the caloric density required to meet the requirements of burn patients.

#### 3.2.3. Protein

Providing adequate amounts of protein post burn is essential as protein stores are depleted for energy usage and muscle tissue is broken down at rates of up to 150 g/day [18]. The nitrogen losses of severely burned patients can amount to 20–25 g/m2TBSA/day [19] leading to detrimental muscle loss and in children a significant decline in growth trajectory for a year or longer post burn [1]. It is also associated with decreased immune function and delayed wound healing. This catabolic state can last upwards of two years leading to gross negative nitrogen balance in the absence of adequate exogenous protein sources. While the goal of protein provision to promote anabolism is unrealistic in the days and weeks immediately following injury, mitigating the catabolic response is both realistic and achievable. 

According to long established research, protein provided at 1.5–2.5 g/kg/day is estimated to be sufficient for mitigating the hypercatabolic response in adults, while 2.5–4.5 g/kg/day in children should be sufficient [1]. Due to these relatively high amounts, enteral nutrition formulas will need to have approximately 25% of their kilocalories derived from protein. The enteral nutrition formula that is used in the Ross Tilley Burn Centre contains just 18% of total kilocalories from protein. Therefore additional supplementation of protein is required to meet the recommendations made for both grams of protein per kilogram of body weight and the appropriate macronutrient distribution of 25% of total kilocalories from protein. This additional protein is provided in the form of a protein powder delivered via the feeding tube.

#### 3.2.4. Conditionally Essential Amino Acids, Alanine and Glutamine

Supplementation of these amino acids has become common in enteral nutrition products aimed at the burn population as both alanine and glutamine become conditionally essential after a burn injury. These enteral products often site the immune enhancing and wound healing properties of these two amino acids. 

In the case of alanine, while it may have a role in wound healing, there are multiple downsides that make it unadvisable for supplementation. Alanine is known to increase the rate of urea production and leads to increased excretion of nitrogen from the body [20]. Its role in the nitric oxide pathway makes it particularly detrimental to burn patients who are septic or at risk of becoming septic [21]. 

In contrast, several studies support the use of enteral glutamine supplements in the adult burn population. Glutamine aids in reducing oxidative stress via its function as a precursor to glutathione. Glutamine is also a source of fuel for macrophages, fibroblasts and lymphocytes and aids in preventing bacterial translocation via preservation of gut integrity by acting as a fuel source for enterocytes [22]. Research has also shown that glutamine supplementation is favourable as it has the potential to decrease length of stay and associated costs through improving wound healing and decreasing rates of infection and mortality [23]. The greatest challenge with glutamine is determining the best route by which to provide it so that it is taken in intracellulary where it has the most effect.

Current literature supports enteral supplementation at 0.3–0.5 g/kg for 14–21 days post burn for patients with greater than 20%–30% TBSA burns [23]. Our centre is currently participating in the multi-centre Re-energize trial to determine the best practice for glutamine supplementation in the adult burn population. 

Currently, the practice in the Ross Tilley Burn Centre includes administration of glutamine in 10 g doses via feeding tube from two to four times daily depending on estimated individual requirements using 0.3–0.5 g/kg/day. Patients with greater than 30% TBSA burns are provided with doses closer to 0.5 g/kg/day, while patients with less than 30% TBSA burns are provided with amounts closer to 0.3 g/kg/day.

### 3.3. Antioxidants

Severely burned patients incur significant oxidative stress due to both the injury and fluid resuscitation early in the treatment process. In order to protect organs against this oxidative stress, antioxidants that scavenge free radicals or inhibit their formation need to be provided to the burn patient [24]. Antioxidant therapies including: ascorbic acid; glutathione; *N*-acetyl-L-cysteine; vitamins A, C and E; alone or in combination have been previously shown to protect microvascular circulation, mitigate changes in cellular energetics, decrease tissue lipid peroxidation and decrease the volume of fluid required for resuscitation [24]. 

Following a burn injury patients have been found to have decreased levels of vitamins A, C, E and D as well as decreases in the trace elements iron, copper, selenium and zinc [1]. Adverse effects resulting from these lower levels include: decreased immune function, poorer wound healing, and decreases in neuromuscular function [1]. The American Society of Enteral and Parenteral Nutrition recommends that burn patients be provided with antioxidant vitamins and trace minerals (specifically selenium) but suggested that additional studies would be required to determine the optimal route, dosage and combination of these nutrients [25]. It has been suggested in a recent systematic review and meta-analysis of antioxidant use in the critical care population that there may be some benefit in administering antioxidants both parenterally and enterally to maximize their effects [26]. 

#### Vitamins A, C and E

Vitamins A, C and E are important in decreasing oxidative stress and the provision of these vitamins as a part of the nutrition prescription can result in the prevention of unnecessary tissue damage [1]. Vitamin A is involved in epithelial growth and may be able to decrease time associated with wound healing [1]. Vitamin C has an active role in the cross-linking and synthesis of collagen [1].

Vitamins C and E have been increasingly studied for their role in buffering the oxidative stress associated with burn injury. Serum levels of vitamin C can decrease by as much as half following a burn injury while serum levels of alpha tocopherol are noted to be depleted by weeks two and three following burn injury [27]. Recent findings have shown that supplementation of vitamin E and C along with zinc has a number of beneficial effects in the pediatric burn population. Children supplemented with vitamin E alone demonstrated decreased levels of lipid peroxidation when compared to children who did not receive an antioxidant supplementation containing vitamin E, vitamin C and zinc [28]. These children also had faster wound healing.

Current practice in the Ross Tilley Burn Centre is to supplement patients with burns greater than or equal to 30% TBSA, or greater than or equal to 20% TBSA full thickness, or greater than or equal to 20% TBSA and intubated with 500 mg of ascorbic acid and 400 units of vitamin E twice daily by mouth or enteric feeding tube. Vitamin A is supplemented through the provision of a multiple vitamin for a total of 3300 IU daily. Patients with smaller burns receive 500 mg ascorbic acid twice daily by mouth or enteric feeding tube along with a multiple vitamin containing 3300 IU vitamin A, 10 IU vitamin E and an additional 100 mg of ascorbic acid.

### 3.4. Trace Elements

Copper, iron, selenium and zinc are trace elements that play and important role in the recovery of burn patients. In burns, early copper, selenium and zinc supplementation for 8–14 days has been associated with a reduction of lung infectious complications, and antioxidant supplementation has also been linked to shorter duration of mechanical ventilation and ICU stay [29]. Copper is required for wound healing through its role in collagen synthesis [28]. Iron is a co-factor in oxygen carrying proteins and is required to ensure adequate oxygenation of tissues. Selenium is important for cell-mediated immunity while zinc is involved with DNA replication, lymphocyte function and protein synthesis [1].

#### 3.4.1. Copper

Copper plays an integral role in wound healing. Adequate levels of copper are required for the initiation of collagen and elastin cross-linking through the extracellular enzyme lysyl oxidase [30]. Wound exudate and urinary losses can lead to a 20–40 percent decrease in the body’s normal copper stores one week post burn [31]. Copper deficiencies have been associated with poor outcomes due to altered immunity and fatal arrhythmias [1]. Current practice in the Ross Tilley Burn Centre includes copper supplementation along with other trace elements.

#### 3.4.2. Selenium

Selenium is important trace element due to its role in activating glutathione peroxidase, a powerful antioxidant, along with being a constituent part of many other seleneoproteins [32]. Following a severe burn injury, patients lose significant amounts of selenium through wound exudate [32]. Selenium deficiency is linked with immune compromise and given that burn patients are prone to nosocomial infection it is important to ensure that they receive supplemental selenium during their course in hospital [32,33]. Burn patients who are supplemented with selenium have been found to have a significantly shorter length of stay in the intensive care unit [32].

Current practice in the Ross Tilley Burn Centre is to supplement patients with burns greater than or equal to 30% TBSA or greater than or equal to 20% TBSA full thickness or greater than or equal to 20% TBSA and intubated with 1000 μg of selenium daily via parenteral route for the first 14 days of admission and then provide 200 μg twice daily by mouth or enteric feeding tube. For those patients with smaller burns no additional selenium is provided beyond that found in a multiple vitamin tablet that is provided once daily.

#### 3.4.3. Zinc

Zinc has many roles within the body including: immune function, DNA synthesis, protein and collagen synthesis, cellular proliferation, and wound healing, cross-linking collagen, stimulating bone formation/mineralization [1,34]. During the first week following a burn injury the combined losses of zinc through urine and wound exudate accounts for roughly 5–10 percent of the normal body content [31]. This depletion of zinc stores and the resultant decreased serum zinc levels appears to be uncorrelated with the size of the burn injury [35]. Within rat models zinc depletion elicited greater levels of burn induced insulin resistance with associated protein catabolism and hyperglycemia than that seen in rats with normal zinc levels [36]. Studies examining the critical care population have found an inverse relationship between serum zinc levels and Sequential Organ Failure Assessment scores [37].

A recent study looking at Canadian burn patients with burn sizes ranging from 10 to 93 percent TBSA found that the enteral supplementation of 50 mg of zinc daily resulted in the normalization of serum zinc levels by discharge in 82 percent of patients [38]. It was also found that this level of enteral zinc supplementation does not interfere with copper levels when administered with a multiple vitamin and micronutrient supplement [38].

Current practice in the Ross Tilley Burn Centre is to supplement patients with burns greater than or equal to 30% TBSA, or greater than or equal to 20% TBSA, full thickness or greater than or equal to 20% TBSA and intubated with 30 mg elemental zinc daily via intravenous for 5 days and then provide 50 mg elemental zinc daily enterally from day 6 onwards. Patients who do not fall under one of the above criteria are provided with 50 mg of zinc daily either by mouth or enteric feeding tube.

### 3.5. Age

The Ross Tilley Burn Centre admits only those over the age of 16 years. As an adult burn centre it regularly admits elderly burn patients. These patients present with their own unique set of nutritional challenges. The elderly burn patient is more likely to have multiple comorbidities including organ dysfunction (*i.e.*, chronic renal failure or congestive heart failure) and an already compromised immune system [39]. Due to the socioeconomic status of many elderly patients they are also more likely to have preexisting protein energy malnutrition prior to their burn injury that further compromises their health status, impedes wound healing, their overall recovery and often leads to poorer outcomes if not properly addressed [39]. When assessing the elderly for enteral nutrition support it is important to be mindful of their decreased basal metabolic requirements [39] as well as any preexisting organ dysfunction that may impair their ability to adequately utilize the nutrients being provided such as in the case of providing high amounts of protein to a patient with preexisting grade 4 renal failure.

### 3.6. Monitoring and Assessment

Assessing and continually reevaluating the actual caloric needs of the burn patient is important for the prevention of overfeeding. While patients are in a hypermetabolic state, providing carbohydrate, fat and protein in excessive amounts can result in complications. The provision of excess carbohydrate can lead to deposition of fat in the liver and results in increased fat synthesis, and difficulties in weaning ventilator support due to elevated respiratory quotients and increased carbon dioxide production. The provision of excess fat leads to further accumulation in the liver and in the case of excessive omega six fatty acids, a proinflammatory response. Excessive amounts of protein can lead to acute renal failure, as a result of increased blood urea nitrogen levels, leading to the need for renal replacement therapy and an increased risk of death [40]. Overfeeding in general can cause problems including hyperglycemia and difficulties weaning from the ventilator due to the retention of carbon dioxide and azotemia without having any greater effect on attenuating the hypermetabolic state than if just adequate amounts of carbohydrate, lipid and protein are provided [1,34]. 

At the Ross Tilley Burn Centre our respiratory therapists perform metabolic cart studies on all ventilated patients every Tuesday afternoon. The registered dietitian then interprets the results of these studies and enteral feeds are adjusted accordingly. The registered dietitian also performs regular assessments and adjusts the enteral nutrition and supplemental protein and glutamine prescription based on multiple patient specific parameters including: patient tolerance to feeds, biochemical changes in bloodwork, weight changes and status of wound healing. Enteral nutrition support is discontinued only once the patient has demonstrated through that they can adequately meet their nutritional requirements by mouth.

## 4. Conclusions

Nutrition is a critical aspect of burn care greatly affecting burn outcomes. Enteral nutrition support remains the best way to address the hypermetabolic, hypercatabolic state of the burn patient. The early provision of nutrition support within the first few hours of admission continues to prove beneficial in the literature. 

There is still no conclusive evidence that mortality rates can be lowered by providing a high carbohydrate, high protein, low fat enteral feed *vs.* a high fat, high protein, low carbohydrate enteral feed there is evidence that providing too much or too little of a micronutrient can have a detrimental effect. Our goal is to provide enteral nutrition support with a macronutrient ratio of 25:50:25, protein:carbohydrate:lipid with a supplement of 0.3–0.5 g/kg of glutamine. 

Evidence continues to show that the provision of omega three fatty acids can be beneficial in terms of immunity, decreasing the inflammatory response and preventing essential fatty acid deficiencies. Conversely, omega six fatty acids continue to be implicated as pro-inflammatory agents when provided in large quantities. Findings related to protein have remained relatively unchanged from a macronutrient perspective, however, as a conditionally essential amino acid alanine has fallen out of favour and there is growing support for supplementing glutamine. 

The research into antioxidants has expanded substantially in recent years with focus in particular on the best way to provide these substances. At this point in time, it would appear that providing antioxidants both enterally and parenterally maximizes their beneficial effects. Vitamin C and E continue to be examined for their role in decreasing lipid peroxidation and wound healing. Zinc has been examined in depth and shows to be a very important component in the recovery process following a burn. Selenium and its associated function of activating glutathione peroxidase is also being studied more closely within the burn population. 

Novel approaches to nutritional components such as antioxidants, glutamine and omega fatty acids will be needed to further improve post burn morbidity and mortality. At present it appears that early enteral nutrition using a high carbohydrate, high protein, low fat enteral solution and the provision of glutamine and an antioxidant cocktail is the best way to address the hypermetabolic state of the burn patient. More research is needed into how to best deliver each of these components and in what amount they need to be supplemented for the burn population. 
